# Socioeconomic - related inequalities in overweight and obesity: findings from the PERSIAN cohort study

**DOI:** 10.1186/s12889-020-8322-8

**Published:** 2020-02-11

**Authors:** Farid Najafi, Shahin Soltani, Behzad Karami Matin, Ali Kazemi Karyani, Satar Rezaei, Moslem Soofi, Yahya Salimi, Mehdi Moradinazar, Mohammad Hajizadeh, Loghman Barzegar, Yahya Pasdar, Behrooz Hamzeh, Ali Akbar Haghdoost, Reza Malekzadeh, Hossein Poustchi, Sareh Eghtesad, Azim Nejatizadeh, Mahmood Moosazadeh, Mohammad Javad Zare Sakhvidi, Farahnaz Joukar, Seyed Mohammad Hashemi-Shahri, Alireza Vakilian, Ramin Niknam, Elnaz Faramarzi, Ghodrat Akhavan Akbari, Fershteh Ghorat, Arsalan Khaledifar, Davoud Vahabzadeh, Reza Homayounfar, Ali Reza Safarpour, Sayed Vahid Hosseini, Reza Rezvani, Seyyed Ahmad Hosseini

**Affiliations:** 1 Research Center for Environmental Determinants of Health, Health Institute, Kermanshah University of Medical Sciences, Kermanshah, Iran; 2 Social Development and Health Promotion Research Center, Kermanshah University of Medical Sciences, Kermanshah, Iran; 3 School of Health Administration, Faculty of Health, Dalhousie University, Halifax, Canada; 4 Modeling in Health Research Center, Institute for Future Studies in Health, Kerman University of Medical Sciences, Kerman, Iran; 5 Liver and pancreatobiliary Diseases Research Center, Digestive Diseases Research Institute, Tehran University of Medical Sciences, Tehran, Iran; 6 Molecular Medicine Research Center, Hormozgan University of Medical Sciences, Bandar Abbas, Iran; 7 Health Sciences Research Center, Addiction Institute, Mazandaran University of Medical Sciences, Sari, Iran; 8 Occupational Health Research Centre, School of Public Health, Shahid Sadoughi University of Medical Sciences, Yazd, Iran; 9 Gastrointestinal and Liver Diseases Research Center, Guilan University of Medical Sciences, Rasht, Iran; 10 Health Promotion Research Center, Zahedan University of Medical Sciences, Zahedan, Iran; 11 Dept. of Neurology, Medical School, Rafsanjan University of Medical Sciences, Rafsanjan, Iran; 12 Gastroenterohepatology Research Center, Shiraz University of Medical Sciences, Shiraz, Iran; 13 Liver and Gastrointestinal Diseases Research Center, Tabriz University of Medical sciences, Tabriz, Iran; 14 Digestive Disease Research Center, Ardabil University of Medical Sciences, Ardabil, Iran; 15 Traditional and Complementary Medicine Research Center, Sabzevar University of Medical Sciences, Sabzevar, Iran; 16 Modeling in Health Research Center, Shahrekord University of Medical Sciences, Shahrekord, Iran; 17 Social Determinants of Health Research Center, Urmia University of Medical Sciences, Urmia, Iran; 18 Noncommunicable Diseases Research Center, Fasa University of Medical Sciences, Fasa, Iran; 19 Colorectal Research Center, Shiraz University of Medical Sciences, Shiraz, Iran; 20 Department of Nutrition, Faculty of Medicine, Mashhad University of Medical Sciences, Mashhad, Iran; 21 Nutrition and Metabolic Diseases Research Center, Ahvaz Jundishapur University of Medical Sciences, Ahvaz, Iran

**Keywords:** Socioeconomic Factors, Inequality, Concentration index, overweight and obesity, PERSIAN, Iran

## Abstract

**Background:**

Overweight and obesity are major health concerns worldwide, with adverse health consequences during the life span. This study measured socioeconomic inequality in overweight and obesity among Iranian adults.

**Methods:**

Data were extracted from 129,257 Iranian adults (aged 35 years and older) participated in the Prospective Epidemiologic Research Studies in IrAN (PERSIAN) in 14 provinces of Iran in 2014. Socioeconomic-related inequality in overweight and obesity was estimated using the Concentration Index (C_n_). The C_n_ further decomposed to find factors explaining the variability within the Socioeconomic related inequality in overweight and obesity.

**Results:**

Of the total number of participants, 1.98, 26.82, 40.76 and 30.43% had underweight, normal weight, overweight and obesity respectively. The age-and sex standardized prevalence of obesity was higher in females than males (39.85% vs 18.79%). People with high socioeconomic status (SES) had a 39 and 15% higher chance of being overweight and obese than low SES people, respectively. The positive value of C_n_ suggested a higher concentration of overweight (0.081, 95% confidence interval [CI]; 0.074–0.087) and obesity (0.027, 95% CI; 0.021–0.034) among groups with high SES. There was a wide variation in socioeconomic-related inequality in overweight and obesity rate across 14 provinces. The decomposition results suggested that SES factor itself explained 66.77 and 89.07% of the observed socioeconomic inequalities in overweight and obesity among Iranian adults respectively. Following SES, province of residence, physical activity, using hookah and smoking were the major contributors to the concentration of overweight and obesity among the rich.

**Conclusions:**

Overall, we found that overweight and obesity is concentrated among high SES people in the study population. . Accordingly, it seems that intersectional actions should be taken to control and prevent overweight and obesity among higher socioeconomic groups.

## Background

Obesity is one of the major health concerns worldwide affecting approximately all physiological roles of the body. It increases the risk for multiple chronic conditions, such as cardiovascular disease [1, 2], diabetes mellitus [1], different kinds of cancers [3], some musculoskeletal disorders [4], and poor mental health [5]. Also, studies show that obesity can have negative influences on the quality of life, healthcare costs and work productivity [6, 7]. The World Health Organization (WHO) has estimated that obesity affects 500 million people worldwide and it could potentially increase to one billion people globally by 2030 [8, 9].

Although overweight and obesity result from a combination of causes, over-consumption of high-energy foods is considered as the primary cause of obesity [10]. In addition, evidence showed that factors such as lack of physical activity, lack of sleep, sedentary lifestyle and high level of stress could also increase the risk of obesity [11, 12]. Iindividual, social and behavioral determinates of obesity [13, 14] may increase the risk of obesity conjointly or independently. For example, an obesogenic behavior like lack of physical activity may be influenced by individual and social factors such as genetic, biological, marital, educational and occupational factors [15, 16].

The existing literature repeatedly has investigated the effects of education and income, as indicators for socioeconomic status (SES), on obesity in both developed and developing countries. Some studies suggested that low education and income can put individuals at the risk of obesity in developed countries [17, 18]. For example, findings of a study in Germany showed that less-educated and low-income people tended to be more obese than their respective counterparts [19]. In contrast, systematic reviews of the current studies revealed a strong positive association between SES and obesity in countries with low human development index (HDI) for both men and women [20]. Accordingly, the study by Dinsa et al. notes that higher educational attainment increases the probability of obesity among the general population [17].

To date, several studies have been conducted to investigate the socioeconomic determinants of obesity, including education, income, occupational status and place of residence in an Iranian population. The results of available studies show consistent findings of the association between socioeconomic factors and overweight and obesity [21–23]. For instance, Bakhshi et al. in a national health survey found that higher education and active workforce decrease the odds of obesity and in contrast higher income and urbanization increase the risk of obesity among both Iranian males and females [22]. Also, Kolahi et al. in a nationwide survey in 31 provinces of Iran found that the socioeconomic factors such as urbanization, living alone, being housewife or retired, and having lower education were associated with overweight and obesity [24].

Although the current studies assessed the effect of different socioeconomic factors on overweight and obesity in Iran, there are limited numbers of studies [25, 26] aimed to quantify socioeconomic inequalities in overweight and obesity in certain provinces of Iran. Accordingly, the main aim of the present study was to measure socioeconomic inequalities in overweight and obesity and then to identify the major contributors to the measured inequality in the outcome variables. Additionally, using the Prospective Epidemiologic Research Study in IRaN (PERSIAN Cohort Study), we identified the risk factors of, and geographical differences in overweight and obesity among adults in 14 provinces in Iran.

## Method

### Data source and variables

Data were extracted from the Prospective Epidemiological Research Studies in IrAN (PERSIAN), which collects epidemiological information from 17 cohort centers in 14 provinces in Iran since 2014. The cohort population for each province has been presented in Table 3. Other detailed information on the cohort method can be found in the past studies [27, 28].

According to statistical census center in 2016, the population of Iran was 79,926,270. Iran has 31 provinces and located in western Asia with a total area of 1,648,195 km^2^. Our study population approximately included 0.16% of Iran population.

Overall 129,257 Iranian adults aged 35 to 70 years participated in this cohort study. The mean age of the cohort population was 49.41 years ±9.18. In all study provinces, participants were recruited from urban settings and entered in the study by multistage cluster sampling. Following identifying households in each cluster, all individuals aged 35 to 70 years who lived in a household included in the study according to the inclusion and the exclusion criteria.

The inclusion criteria in the cohort study included:

1. General population aged 35 to 70 years
2. Households located within the study area
3. People with Iranian nationality

Also, exclusion criteria included:

1. People who were reluctant to participate in the study.
2. People with communication disorders who were not able to answer the study questions
3. People with hearing and intellectual disability, mental disorders, and vision loss.

In the study, the outcome variables were a binary variable indicating whether the participant had overweight (Body Mass Index (BMI) = 25–29.9 kg/m2) and obesity (BMI ≥ 30 kg/m2) [29]. Also, BMI less than 18.5 and 18.5–24.9 were classified as underweight and normal weight respectively. Several sociodemographic (age, sex, marital status), socioeconomic, behavioral (physical activity, cigarette smoking, hookah smoking, alcohol consumption and drug abuse) and geographical factors were used as determinants of overweight and obesity in the analysis. Physical activity was measured on a weekly basis using Metabolic Equivalent Rates (METs) of self-reported daily activities of participants. One MET is equal to resting metabolic rate, the amount of oxygen consumed at rest that is about 3.5 ml of oxygen per kilogram per minute. Given that four METs requires 16 ml oxygen/kilogram/minute [30], MET of each activity were extracted using compendium of physical activities [31, 32]. With regard to the mean MET rates of participants (41 METs/hour/day), participants with less than 41 METs/hour/day were defined as individuals with poor physical activity level. In the present study, alcohol consumers were individuals who used at least 12 drinks in the past year [33]. Also, drug abuse was defined as lifetime use of any kinds of illicit drugs (e.g. opium, heroin, cocaine, crack, etc.).

Concerning the definition of smoking provided in the National Health Interview Survey (NHIS), current smokers were individuals who smoked 100 cigarettes in their lifetime and who currently smoke cigarettes regularly. The former smokers were defined as people who have quit cigarette and/or tobacco use [34]. Furthermore, Hookah (water pipe used to smoke flavored tobacco) smoking was defined as at least one session per month [35]. In the cohort study, the variables of drug abuse, alcohol consumption and hookah smoking were measured by a self-report questionnaire.

Regarding the difficulties in estimating SES with income and consumption, past studies have focused on developing proxy indicators. In global health research, the wealth index has been proposed as one of the most important key proxy indicators [36]. For example, some researchers have applied the wealth index to investigate subjects such as malnutrition [37], prenatal care [38], malaria transmission [39], reproductive health [40], and poverty [41].

In the present study, given available data, we used information on assets ownership (e.g., owning car, motorcycle, bicycle, refrigerator, stove, vacuum machine, personal computer, sewing machine and washing mashing), housing characteristics (e.g. bathroom, house area per capita) and education level of participants to create SES variable. Therefore, the SES indicator was constructed by a combination of households’ assets and education levels of participants following a procedure developed by Filmer and Pritchett [42] based on principal components analysis (PCA). In this study, this method was used to reduce multi-dimensional data sets on ownership of different household assets to a lower number of dimensions.

As socioeconomic status (SES) was an important determinant to measure inequality in overweight and obesity, we performed the principal component analysis (PCA) to construct a rank variable when we measured socioeconomic-related inequality [43]. Participants were categorized into five SES quintile from the lowest (1st quintile) to highest (5th quintile) SES groups.

### Statistical analysis

#### Socioeconomic-related inequality in overweight and obesity

The Concentration index (Cn) measures inequality in the outcome variable (e.g. obesity) over the distribution of an explanatory variable (e.g. socioeconomic status). In fact, the concentration index indicates the extent to which our outcomes (overweight and obesity) differs across individuals ranked by SES [44]. The C_n_ is based on the concentration curve which graphs the cumulative percentage of a population according to their SES on the horizontal axis and the cumulative percentage of health outcome (overweight and obesity) on the vertical axis. The C_n_ is twice the area between the concentration curve and line of perfect equality (the 45-degree diagonal line). The value of the C_n_ varies between − 1 and + 1. The numbers − 1 and + 1 show the highest socioeconomic related inequality among a population. The negative value of the C_n_ suggests the concentration of the health outcome among the poor and vice versa. The zero value of the C_n_ reveals equal socioeconomic distribution of the health outcome among the different SES groups. The C_n_ can be measured using following “convenient covariance” formula [45]:

1 $$ c=\frac{2\ast co\upsilon \left({y}_i\;{r}_i\right)}{\mu } $$

where *y*_*i*_ is health outcome variable (i.e., overweight and obesity) for participant *i*, *r*_*i*_ is the fractional rank of participant *i* in the distribution of SES indicator, *μ* is the mean of the health outcome variable. As overweight and obesity is a binary variable, the minimum and maximum of the C are not − 1 and + 1. Thus, as per Wagstaff suggestion [46], we normalized the C_n_ as:

2 $$ {C}_n=\frac{1}{1-\mu } $$

### Decomposition of socioeconomic inequality in overweight and obesity

The estimated value of the normalized C_n_ was decomposed to identify the contribution of explanatory variables to the observed socioeconomic inequality in overweight and obesity [47]. Wagstaff and colleagues [47] noted that if we have a regression model relating a health outcome variable of *y* to a set of *k* explanatory variables, *x*, such as:

3 $$ y=a+\sum \limits_k{\beta}_k\;{\chi}_k+\varepsilon, $$

the C_n_ for *y* can be decomposed as:

4 $$ C=\sum \limits_k\left(\frac{\beta_k\overline{\chi_k}}{\mu}\right)\;{C}_k+G\;{C}_{\varepsilon }/\mu . $$

In this equation, ${\overline{x}}_k$ denotes the mean of the explanatory variable, *x*, *C*_*k*_ is the C_n_ for each explanatory variable, *GC*_*ε*_ is the generalized C_n_ for *ε*. The first component in equation 4, $\sum \limits_k\left(\ \frac{\beta_k{\overline{x}}_k}{\mu}\right){C}_k$ indicates the contribution of explanatory variable *x* to the overall socioeconomic-related inequality in the health outcome. The negative (positive) contribution of an independent variable indicates that the SES-related distribution of this variable and its relation with overweight and obesity increase the concentration of overweight and obesity among the poor (the rich). The second component in equation 4, $\frac{G{C}_{\varepsilon }}{\mu }$ shows the proportion of socioeconomic inequality in overweight and obesity which is not explained by the systematic variation of the included explanatory variables across SES groups. Applying Wagstaff’s correction into Equation [46] yields to:

5 $$ {C}_n=\frac{C}{1-\mu }=\frac{\sum \limits_k\left(\frac{\beta_k{\overline{x}}_k}{\mu}\right){C}_k}{1-\mu }+\frac{G{C}_{\varepsilon }/\mu }{1-\mu } $$

As overweight and obesity is a binary variable, we used marginal effects obtained from a logistic model as *β* in the decomposition of the *C*_*n*_. All the analyses were performed using Stata version 14.2 (StataCorp, College Station, TX, USA).

**Fig. 1:**
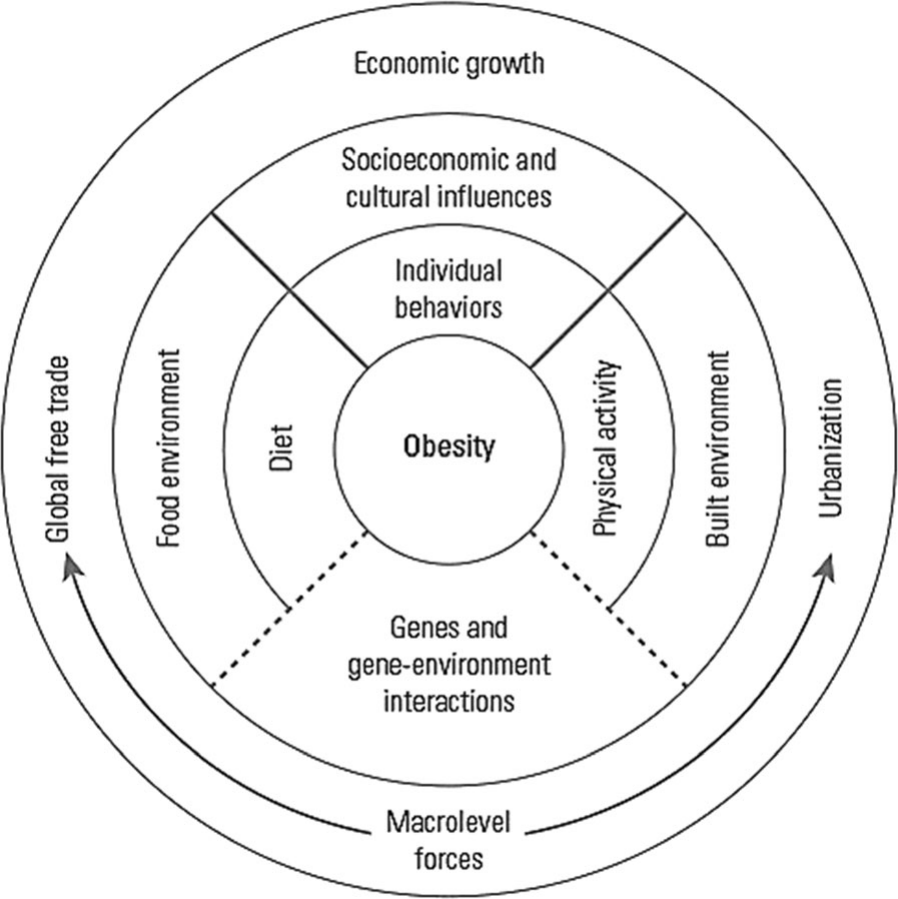
Determinants of obesity (developed by Malik and Hu (2017))

Also, adjusted Odds ratio (OR) with 95% CI was applied to measure the association between the determinants and the outcome variables among the cohort population. Accordingly, the conceptual framework that guided our analysis was developed by Malik and Hu (Fig. 1) [48]. According to available data, we included socioeconomic and cultural factors (age, gender, marital status and SES), individual behaviors (cigarette and hookah smoking, drug abuse, and alcohol consumption) and physical activity. Also, the region of residence was included in our analysis as a macrolevel factor.

## Results

### Prevalence of overweight and obesity

Table 1 reports the crude prevalence, age-and sex-standardized prevalence and adjusted OR for outcome variables by characteristics of participants. The prevalence of underweight, normal weight, overweight and obesity was 1.98, 26.82, 40.76 and 30.43% in the present study respectively. Of the total of 129,257 adults participated in the study, 57,614(44.57%) were males and 71,643(55.43%) were females. Although women had the higher age-standardized prevalence (39.85% vs 18.79%) and odds (OR: 2.83, 95% CI: 2.73–2.92) of obesity than men, they indicated the lower age-standardized prevalence (38.98% vs 42.98%) and odds (OR: 0.78, 95% CI: 0.82–0.86) of overweight compared to men. Participants in the age groups of 35–44 years and 45–54 years had the highest sex-standardized prevalence of overweight (41.47%) and obesity (33.38%) respectively.

**Table 1:** Prevalence of overweight and obesity by characteristics of participants

Variables		Study population (%)	Overweight			Obesity		
			Frequency (crude prevalence)	Age and sex standardized prevalence	Adjusted OR (95% CI)	Frequency (crude prevalence)	Age and sex standardized prevalence	Adjusted OR (95% CI)
Sex	Male	57,614 (44.57)	24,765 (42.98)	42.98*	1.00	10,805 (18.75)	18.79*	1.00
	Female	71,643 (55.43)	27,923 (38.98)	38.95*	0.78 (0.82–0.86)	28,532 (39.83)	39.85*	2.83 (2.73–2.92)
Age groups (years)	35–44	45,809 (35.44)	18,980 (41.43)	41.47**	1.00	13,144 (28.69)	28.41**	1.00
	45–54	43,481 (33.64)	17,578 (40.43)	40.43**	0.98 (0.96–1.01)	14,537 (33.43)	33.38**	1.31 (1.26–1.34)
	55–64	31,573 (24.43)	12,732 (40.33)	40.24**	1.01 (0.98–1.04)	9511 (30.12)	30.55**	1.14 (1.11–1.18)
	> = 65	8394 (6.49)	3398 (40.48)	40.31**	1.04 (0.99–1.09)	2145 (25.55)	26.28**	0.89 (0.84–0.94)
Marital status	Single	2910 (2.25)	1017 (34.95)	37.37	1.00	14,537 (33.43)	16.07	1.00
	Married	117,521 (90.92)	48,368 (41.16)	41.01	1.23 (1.14–1.33)	35,539 (30.24)	30.96	2.61 (2.36–2.89)
	Widowed and divorced	8826 (6.83)	3303 (37.42)	37.91	1.19 (1.09–1.3)	3304 (8.4)	27.39	2.54 (2.28–2.84)
Smoking	No	101,136 (78.24)	41,779 (41.31)	42.27	1.00	34,134 (33.75)	31.02	1.00
	Current smoker	18,115 (14.01)	6522 (36)	34.21	0.73 (0.7–0.76)	2841 (15.68)	24.03	0.68 (0.65–0.72)
	Former smoker	10,006 (7.74)	4387 (43.84)	40.14	0.99 (0.94–1.03)	2362 (23.61)	32.01	1.07 (1.02–1.13)
Physical activity	Good	52,681(40.75)	21,292 (40.42)	40.32	1.00	13,928 (26.44)	27.69	1.00
	Poor	76,576(59.25)	31,396 (40.99)	41.30	1.02 (0.99–1.04)	25,409 (33.18)	32.63	1.35 (1.31–1.38)
Hookah smoking	No	114,949 (88.93)	46,579 (40.52)	40.65	1.00	35,602 (30.97)	30.11	1.00
	Yes	14,308 (11.07)	6109 (42.7)	40.11	0.73 (0.71–0.76)	3735 (26.1)	32.45	1.28(1.23–1.34)
Drug abuse	No	113,812 (88.05)	47,221 (41.49)	41.87	1.00	36,631 (32.19)	30.98	1.00
	Yes	15,445 (11.95)	5467 (35.4)	34.86	0.73 (0.71–0.76)	2706 (17.52)	29.66	0.91 (0.87–0.96)
Alcohol consumption	No	117,559 (90.95)	47,690 (40.57)	40.78	1.00	37,011 (31.48)	30.38	1.00
	Yes	11,698 (9.05)	4998 (42.73)	42.81	1.14 (1.09–1.19)	2326 (19.88)	27.69	1.05 (1.01–1.11)
Provinces	Kermanshah	10,040 (7.77)	4355 (43.38)	43.37	1.00	2710 (26.99)	27.15	1.00
	Hormozgan	3285 (2.54)	1278 (38.9)	38.95	0.82 (0.76–0.89)	786 (23.93)	23.15	0.77 (0.71–0.84)
	Fars	22,939 (17.75)	8927 (38.92)	39.04	0.93 (0.88–0.97)	13,165 (18.47)	18.61	0.61 (0.57–0.64)
	Sistan and Baluchestan	8152 (6.31)	3100 (38.03)	37.53	0.81 (0.75–0.85)	2234 (27.4)	26.46	0.85 (0.79–0.91)
	Razavi Khorasan	2868 (2.22)	1333(46.48)	46.82	0.97 (0.89–1.06)	619 (21.58)	23.58	0.73 (0.65–0.81)
	Kerman	9885 (7.65)	4061 (41.08)	41.11	0.89 (0.84–0.95)	2978 (30.13)	30.46	1.08 (1.01–1.15)
	Chaharmahal and Bakhtiari	6655 (5.15)	3101(46.6)	46.12	1.02 (0.96–1.09)	1866 (28.04)	29.03	1.01 (0.94–1.09)
	Mazandaran	10,252 (7.93)	4348 (42.41)	42.75	0.92 (0.87–0.97)	3430 (33.45)	32.57	1.35 (1.26–1.43)
	Guilan	10,494 (8.12)	4192 (39.95)	39.89	0.91 (0.86–0.96)	3423 (32.62)	33.11	1.34 (1.26–1.43)
	Yazd	9388 (7.26)	3983 (42.43)	41.95	0.93 (0.88–0.99)	3190 (33.98)	35.49	1.41 (1.31–1.49)
	Khouzestan	8991 (6.96)	3319 (36.91)	37.28	0.78 (0.74–0.83)	3497 (38.89)	37.94	1.61 (1.51–1.72)
	West Azerbaijan	3172 (2.54)	1240 (39.09)	39.57	0.88 (0.81–0.96)	1172 (36.95)	35.29	1.66 (1.52–1.81)
	East Azerbaijan	14,958 (11.57)	6130 (40.98)	40.97	0.89 (0.84–0.94)	5629 (37.63)	37.66	1.63 (1.54–1.73)
	Ardabil	8178 (6.33)	3321 (40.61)	40.61	0.84 (0.79–0.91)	3565 (43.59)	43.53	2.05 (1.92–2.19)
Socioeconomic Status	1st quintile (the lowest)	25,995 (20.11)	9509 (36.58)	35.88	1.00	6751 (25.97)	23.57	1.00
	2nd quintile	25,901 (20.04)	10,004 (38.62)	38.61	1.06()1.03–1.11)	7943 (30.67)	29.82	1.25 (1.21–1.31)
	3rd quintile	25,819 (19.97)	10,144 (39.29)	39.31	1.08(1.04–1.12)	8787 (34.03)	33.88	1.41 (1.35–1.47)
	4th quintile	25,778 (19.94)	10,597 (42.51)	42.12	1.21(1.17–1.26)	8583 (33.3)	34.68	1.40 (1.34–1.46)
	5th quintile (the highest)	25,764 (19.93)	12,074 (46.86)	45.75	1,39(1.33–1.45)	7273 (28.23)	31.28	1.15 (1.11–1.21)
Total		129,257 (100)	52,688 (40.76)			39,337 (30.43)		

* Sex comparisons are standardized for age. **Age comparisons are standardized for sex

**Fig. 2:**
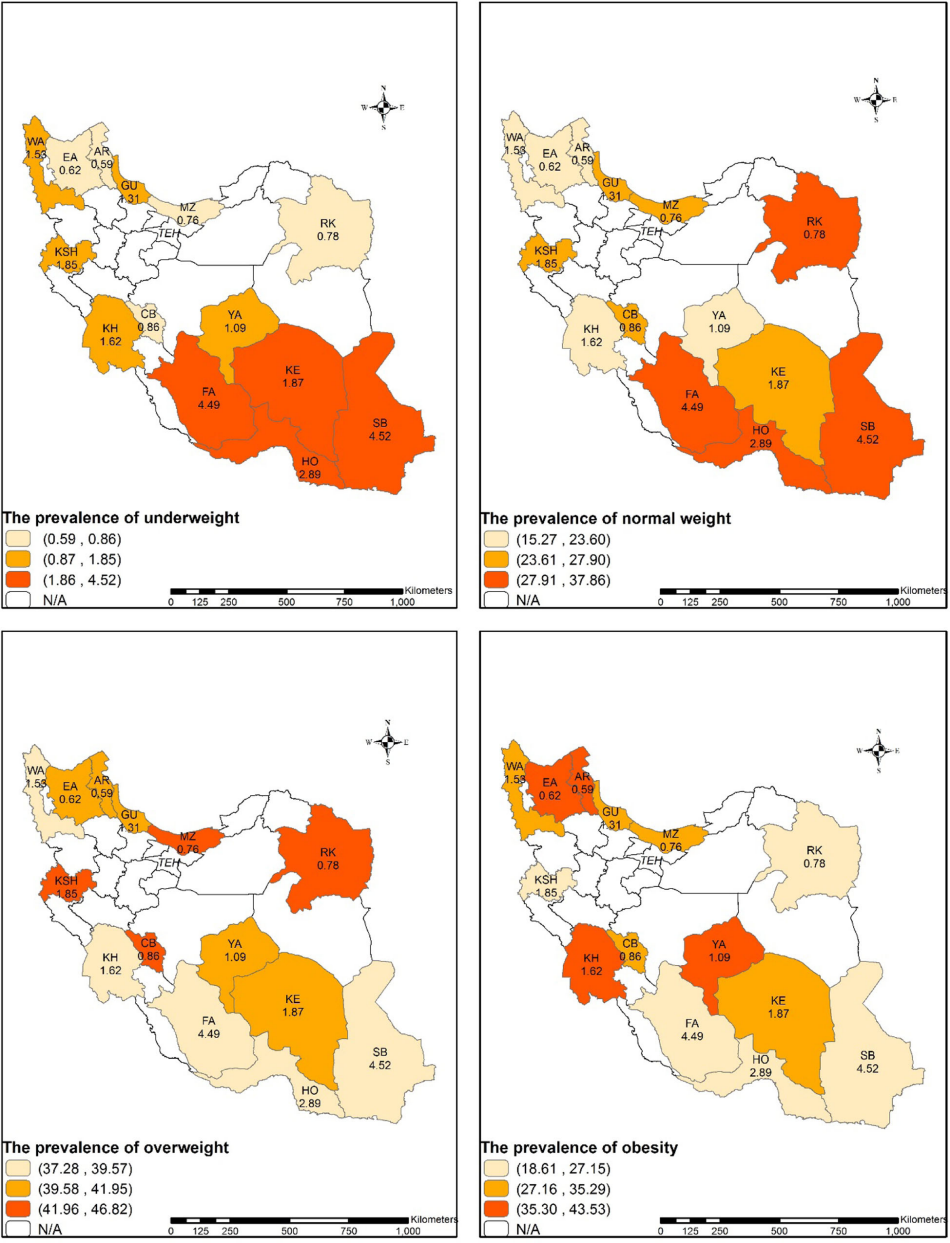
the age-and sex-standardized prevalence of underweight, normal weight, overweight and obesity among the study provinces (Razavi Khorasan(RK), Chaharmahal and Bakhtiari(CB), Yazd(YA), East Azarbaijan(EA), Ardabil(AR), West Azarbaijan(WA), Kerman(KE), Guilan(GU), Hormozgan(HO), Fars(FA), Kermanshah(KSH), Sistan and Baluchestan(SB), Mazandaran(MA), Khouzestan(KH))(developed by the authors using ArcGIS Desktop version 10.7)

The cohort of Ardabil had the highest age-and sex-standardized prevalence (46.82%) and odds (OR: 2.05, 95% CI: 1.92–2.19) of obesity in comparison to other provinces. Although, Razavi Khorasan had the highest age-and sex-standardized prevalence (46.82%) of overweight, the cohort population in Chaharmahal and Bakhtiari had the highest odds of overweight (OR: 1.02, 95% CI: 0.96–1.09) compared to other study provinces. Fig. 2 shows the age-and sex-standardized prevalence of underweight, normal weight, overweight and obesity in the included population.

Regarding Table 1, being married, widowed and divorced (compared to singles), and higher SES significantly increased the probability of overweight among the cohort population. On the other side, being female, being older, being married, widowed and divorced (compared to singles), former smoking, poor physical activity, alcohol consumption and higher SES significantly increased the probability of obesity among participants in the present study.

### Socioeconomic inequalities in overweight and obesity

**Figure 3:**
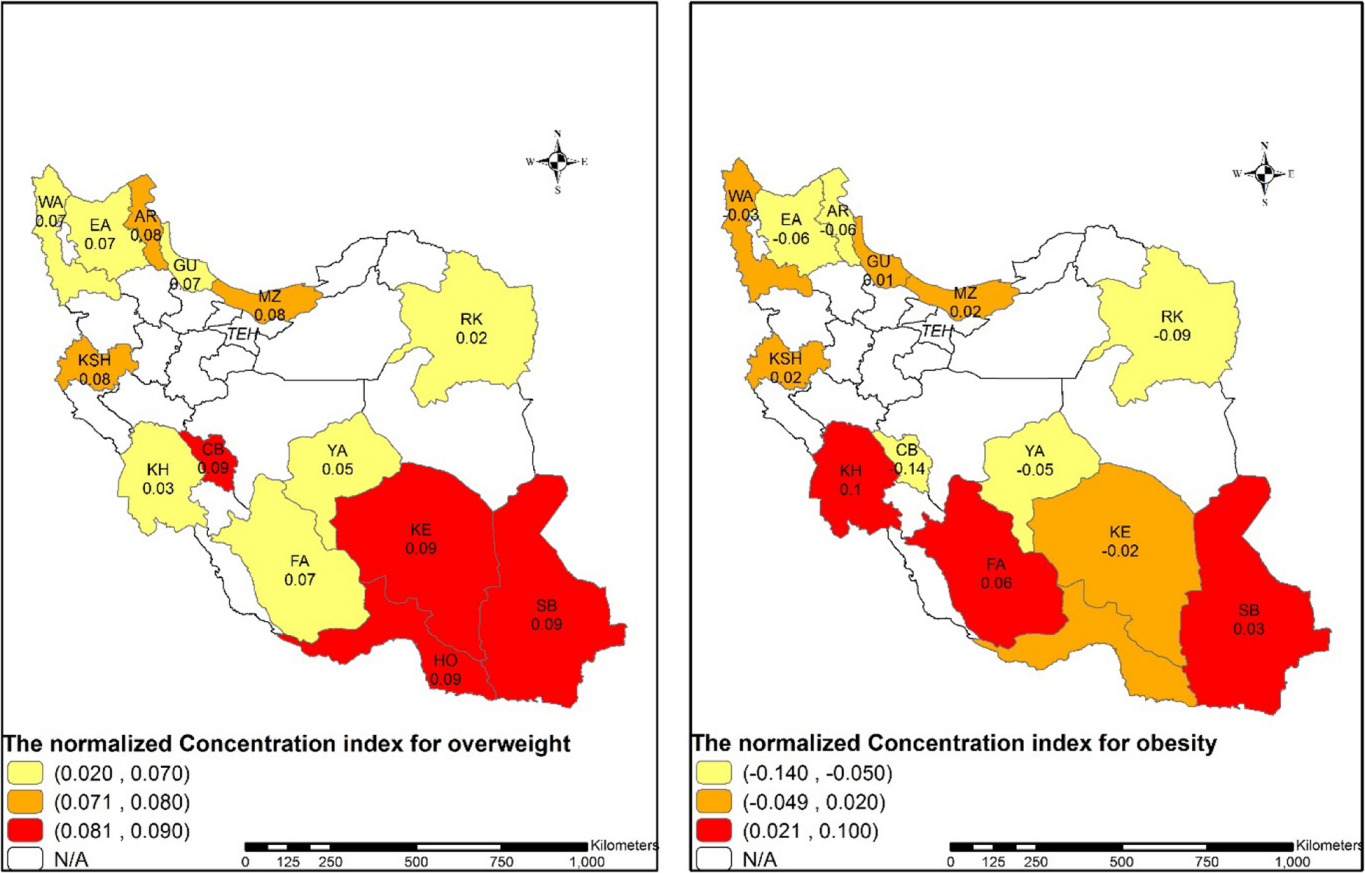
the concentration index for overweight and obesity among the study provinces (Razavi Khorasan(RK), Chaharmahal and Bakhtiari(CB), Yazd(YA), East Azarbaijan(EA), Ardabil(AR), West Azarbaijan(WA), Kerman(KE), Guilan(GU), Hormozgan(HO), Fars(FA), Kermanshah(KSH), Sistan and Baluchestan(SB), Mazandaran(MA), Khouzestan(KH)) (developed by the authors using ArcGIS Desktop version 10.7)

In this study, the positive value of the *C*_*n*_ (*C*_*n*_ = 0.027, 95% CI: 0.021, 0.034) for total provinces indicated the higher concentration of obesity among high-SES adults in the study population. The estimated value of the *C*_*n*_ was positive in 7 provinces and negative in the remaining for 7 provinces. The highest concentration of obesity among the high SES and the low SES groups was observed in Khouzestan (*C*_*n*_ = 0.097, 95% CI: 0.073, 0.121) and Razavi Khorasan (*C*_*n*_ = − 0.087, 95% CI:-0.131, − 0.451) provinces, respectively. Similarly, the C_n_ for overweight was positive which indicate overweight is concentrated among high SES individuals. Fig. 3 illustrates the variation in socioeconomic inequality in overweight and obesity among Iranian provinces.

### Decomposition of socioeconomic inequality in overweight and obesity

Turning to the contribution results reported in Table 2, it is evident that the SES factor explained 66.77 and 89.07% of the overall socioeconomic inequality in overweight and obesity respectively. Following SES, province of residence was the second largest contributor to the concentration of overweight and obesity among the rich and explained 22.62 and 56.19% of the overall socioeconomic inequality in overweight and obesity respectively. Physical activity level had a positive influence on the overall inequality, and it explained 9.42 and 22.74% of the observed inequality in overweight and obesity among the cohort population, respectively. Although gender and age made positive contributions to socioeconomic inequality in obesity, they made a negative contribution to socioeconomic related inequalities in overweight. Also, the decomposition analysis showed that cigarette smoking and hookah use (obesity only) were the main positive contributor to the observed inequality in overweight and obesity among the study population (Table 2).

**Table 2:** Decomposition of socioeconomic inequality in overweight and obesity in the included population

Variables		Overweight				Obesity			
		Elasticity	Concentration index (C_k_)	percentage contribution	Summed Percentage Contribution	Elasticity	concentration Index (C_K_)	percentage contribution	Summed Percentage Contribution
Sex	male				−3.74				3.53
	female	0.082	−0.025	−3.74		0.338	0.006	3.530	
Age	35–44				−1.38				3.29
	45–54	0.028	0.031	1.52		0.061	0.035	4.012	
	55–64	0.013	−0.134	−3.02		0.019	−0.128	−4.471	
	> = 65	0.000	−0.315	0.12		−0.006	−0.317	3.745	
Marital status	single				2.91				4.64
	married	0.329	0.021	12.23		0.565	0.021	22.741	
	others (widow, divorced)	0.019	−0.267	−9.32		0.042	−0.230	−18.106	
Smoking status	never smoked				6.34				9.97
	current smoker	−0.039	−0.091	6.38		−0.048	−0.115	10.396	
	Former smoker	0.001	−0.036	−0.04		0.004	−0.063	−0.426	
Use Hookah	no				2.06				27.89
	yes	0.009	0.123	2.06		0.145	0.103	27.887	
Drug abuse	no				3.75				3.04
	yes	−0.020	−0.104	3.75		−0.014	−0.120	3.043	
Alcohol use	no				1.23				0.93
	yes	0.006	0.112	1.23		0.006	0.088	0.931	
Physical activity (METs/hour/day)		−0.298	−0.017	9.42	9.42	−0.706	−0.017	22.742	22.74
Province	Fars				22.62				56.19
	Guilan	0.003	−0.144	−0.86		0.015	−0.138	−3.871	
	Sistan and Baluchestan	−0.031	−0.360	19.86		−0.056	−0.384	40.266	
	Kermanshah	0.003	−0.063	−0.385		0.026	−0.077	−10.846	
	Chaharmahal and Bakhtiari	0.005	0.157	1.31		0.017	0.163	5.212	
	Mazandaran	−0.012	0.028	−0.62		−0.013	0.022	−0.517	
	Razavi Khorasan	0.004	0.204	1.46		0.015	0.206	5.735	
	Kerman	−0.003	0.285	−1.62		0.002	0.283	0.948	
	West Azarbaijan	0.000	−0.1860	−0.08		0.016	−0.130	−3.987	
	Hormozgan	0.001	0.477	0.83		0.002	0.441	1.353	
	Yazd	−0.005	−0.060	0.58		−0.006	−0.082	0.942	
	Khouzestan	0.002	−0.118	−0.40		0.008	−0.121	−1.749	
	East Azarbaijan	0.010	0.266	4.66		0.031	0.267	15.574	
	Ardabil	−0.003	0.602	−3.07		−0.004	0.602	−5.062	
Socioeconomic status (SES)	1st quintile (the lowest)				66.77				89.07
	2nd quintile	0.022	−0.372	−14.89		0.037	−0.362	−24.913	
	3rd quintile	0.030	0.017	0.91		0.054	0.051	5.136	
	4th quintile	0.038	0.398	27.32		0.060	0.449	50.825	
	5th quintile (the highest)	0.037	0.794	53.43		0.038	0.821	58.022	
Explained					110.00				221.28
Residuals					−10.00				−121.28
Total					100.00				100.00

**Note:** MET = Metabolic Equivalent of Task

## Discussion

Using information derived from the PERSIAN Cohort Study, we analyzed overweight and obesity among Iranian adults aged 35 years and older. Specifically, we measured and decomposed socioeconomic inequalities in overweight and obesity in Iran. Our descriptive results suggested 18.75% of men and 39.83% for women had obesity in the included population. There was also substantial variation in the prevalence of obesity across included provinces.

The results of our study suggested that overweight and obesity were concentrated among the high SES adults in the cohort population as a whole. In agreement with our findings, the previous studies in Iran showed that obesity was less prevalent among low-SES people. For example, Najafi et al. found lower obesity prevalence among poor people in an sample of the Iranian population [25]. Also, Mohammedi et al. (2011) found that income had a positive association with obesity [49]. On the contrary, some studies indicate that people with lower education levels tend to be more obese than their counterparts with higher education [50].

Among the study provinces, Chaharmahal and Bakhtiari and Khouzestan had the lowest and the highest value of C_n_. This finding shows that in Chaharmahal and Bakhtiari and Khouzestan distribution of obesity is disproportionately borne by the low and high SES individuals respectively. This result probably indicates that participants with higher SES in Khouzestan are more likely to have a sedentary lifestyle and an unhealthy dietary pattern in comparison to their counterparts in Chaharmahal and Bakhtiari. Some studies in Khouzestan indicated that lower educational levels, low physical activity, food habits and sedentary lifestyle could be the major causes of obesity in both men and women in Khouzestan [51–53].

Additionally, factors such as cultural, environmental and regional climate diversities presumably can contribute to the observed differences in this socioeconomic inequality. Khouzestan is located in the south-west of Iran and has a hot desert climate [54]. In this province, day time temperatures in most parts reach above 50 °C during dry seasons which in turn can affect the levels of physical activity and dietary patterns among individual with higher SES. In other words, individuals with higher SES might prefer to spend more time at home and have less physically activity compared to their peers in other provinces.

According to our findings, SES, region of residence, physical activity, cigarette smoking, and hookah smoking (only for obesity) were the main positive contributors to socioeconomic-related inequality in overweight and obesity. Consistent with our results, literature in both developed and developing countries indicates that the factors such as income, marital status, education and physical activity were the major factors explaining socioeconomic-related inequality in obesity [25, 55–57]. In contrast, some studies note that factors such as genetic determinants, environment features, race and family history of obesity can explain the concentration of obesity in a population that should be examined in future studies [58–60].

In the present study, SES was the largest contributing factor to the inequality of overweight and obesity. The positive contribution demonstrates that SES has a major role in the disproportionate distribution of overweight and obesity among the study population. Socioeconomic differences can affect the contribution of the variables in the inequality of obesity. For example, in bilger’s et al. (2017) study, Age was the largest contributor to the positive socioeconomic inequality among the participants [59]. Also, they found no socioeconomic inequality in obesity for Mexican Hispanics.

Additionally, region of residence and physical activity were the second and third positive contributors to the socioeconomic inequalities in overweight and obesity among the participants. With respect to these findings, the variation of outcome variables between the study provinces can increase socioeconomic inequality in overweight and obesity. As above mentioned, the result may be due to different lifestyle and dietary habits between the study provinces. Also, the positive contribution of physical activity shows that overweight and obesity are distributed disproportionally between individuals with different levels of physical activity. This finding can show a reverse causality between BMI and physical activity so that as participants gain weight, they tend to become less physically active.

It should be noted that marital status made a positive contribution to socioeconomic inequality in overweight and obesity as well. The finding demonstrates that married adults were more likely to be rich and obese than single individuals. The finding is consistent with the results of other studies in Iran [23, 25, 50, 61–63], which revealed a higher probability of obesity among both married women and men in comparison with single adults. In consistent with our findings, Studies suggested changes in the lifestyle and nutrition patterns after marriage as one of the factors to the higher BMI among adults. For example, the findings of Azadbakht et al. (2005) indicated that the percentage of energy and fat intake was higher among married people compared to single persons [21]. Also, Sartorius et al. (2015) found that single people spent more time exercising compared to married people [64].

In the present study, sex made a negative contribution to socioeconomic inequality in overweight among the participants. This negative contribution is the result of both the negative CI for females and the positive elasticity of all measures of obesity with respect to sex. Similar to previous studies in Iran [65, 66], our results suggested females are more likely to be obese than males. Sedentary lifestyle of women [49, 63, 66, 67] was regarded as one of the main factors contributing to the higher BMI among women in the study population. In the past studies, other factors such as unemployment, depression, unhealthy nutrition patterns, sleep disorders, and illiteracy, low SES, number of pregnancy, and lack of physical activity have identified as the risk factors of obesity among women [68].

Overall, our findings suggest that the burden of overweight and obesity be disproportionately borne by individuals with higher SES. Accordingly, it seems that intersectional actions should be taken to control and prevent overweight and obesity among higher socioeconomic groups. Given that women were more likely to be rich and obese, researchers need to identify the risk factors of obesity among different socioeconomic groups. Because in each group, the risk factors of obesity may be different from the other.

### Limitations

The present study faced some limitations. This study was a cross-sectional analysis of a longtidutional cohort study that shows the measurements only for a time point, not a period. Also, data for all provinces and people under age 35 had not been included in PERSIAN cohort study. Given the positive relationship between age and obesity [69], the nonparticipation of individuals younger than 35 years may result in a higher prevalence of obesity among the cohort population. Third, our findings in the cohort population, may not be representative of the whole population of the study provinces because our data has been collected only in one or two cohort centers in each province. Forth, regarding that the information on drug abuse, alcohol consumption and hookah smoking were measured by a self-report questionnaire, probability of social desirability bias in our reported measures may be unavoidable. Given that obesity derived from a combination of causes and contributing factors, we are not able to have a casual inference in obesity. Different factors such as environment features, ethnic groups, dietary patterns, family history of obesity, and family size may contribute to inequality in obesity that can be investigated in future studies.

**Table 3:** The cohort population in the study provinces

Row	Province	*Population	Cohort site	*Population	Cohort population	Main Ethnicities
1	Ardabil	1,270,420	Ardabil	529,374	8192	Turk
2	Chaharmahal and Bakhtiari	947,763	Sharekord	93,104	6664	Lor
3	East Azerbaijan	3,909,652	Khameneh	3056	14,978	Turk, Azari
4	Fars	4,851,274	Kavar	31,711	2244	Fars (Persian), Turk
			Kharameh	18,477	10,662	Fars (Persian), Arab
			Fasa	110,825	10,113	Fars (Persian), Arab and Turk
5	Guilan	2,530,696	Some’e Sara	58,658	10,511	Gilaki
6	Hormozgan	1,776,415	Bandare Kong	19,213	3570	Arab
7	Kerman	3,164,718	Rafsanjan	161,909	9982	Fars (Persian)
8	Kermanshah	1,952,434	Ravansar	47,657	10,077	Kurd
9	Khouzestan	4,710,506	Hoveizeh	19,481	9156	Arab
10	Mazandaran	3,283,582	Sari	309,820	10,253	Tabari
11	Razavi Khorasan	6,434,501	Mashhad	3,001,184	2189	Fars (Persian)
			Sabzevar	243,700	784	Fars (Persian)
12	Sistan and Balouchestan	2,775,014	Zahedan	587,730	8318	Balouch
13	West Azerbaijan	3,265,219	Ghoushchi	2787	3662	Turk, Azari
14	Yazd	1,138,533	Shahedieh, Yazd	18,309	9901	Fars (Persian)

*The frequency of population is according to Iranian Population and Housing Census in 2016 [70]

## Conclusion

Overall, our results showed that overweight and obesity were concentrated among well-off adults in the study population. Accordingly, it seems that intersectional actions should be taken to control and prevent overweight and obesity among higher socioeconomic groups.

## Data Availability

Data and all other materials for this study are kept at the deputy of research and technology of Kermanshah University of Medical Sciences. The datasets generated and/or analyzed during the current study are not publicly available due the terms of consent to which the participants agreed but are available from the corresponding author on reasonable request.

## Abbreviations

BMI: Body Mass Index;
CI: Confidence Interval
C_n_: Concentration Index
METs: Metabolic Equivalent Rates
NHIS: National Health Interview Survey
PCA: Principal Component Analysis
PERSIAN: Prospective Epidemiologic Research Studies in IrAN
SES: Socioeconomic Status
WHO: World Health Organization
