# Correction: Socioeconomic gradient in physical activity: findings from the PERSIAN cohort study

**DOI:** 10.1186/s12889-022-14060-5

**Published:** 2022-10-19

**Authors:** Farid Najafi, Shahin Soltani, Behzad Karami Matin, Ali Kazemi Karyani, Satar Rezaei, Moslem Soofi, Yahya Salimi, Mehdi Moradinazar, Mohammad Hajizadeh, Loghman Barzegar, Yahya Pasdar, Behrooz Hamzeh, Ali Akbar Haghdoost, Reza Malekzadeh, Hossein Poustchi, Sareh Eghtesad, Azim Nejatizadeh, Mahmood Moosazadeh, Mohammad Javad Zare Sakhvidi, Farahnaz Joukar, Seyed Mohammad Hashemi-Shahri, Alireza Vakilian, Ramin Niknam, Elnaz Faramarzi, Ghodrat Akhavan Akbari, Fershteh Ghorat, Arsalan Khaledifar, Davoud Vahabzadeh, Reza Homayounfar, Ali Reza Safarpour, Sayed Vahid Hosseini, Reza Rezvani, Seyyed Ahmad Hosseini

**Affiliations:** 1 Research Center for Environmental Determinants of Health, Health Institute, Kermanshah University of Medical Sciences, Kermanshah, Iran; 2 Social Development and Health Promotion Research Center, Kermanshah University of Medical Sciences, Kermanshah, Iran; 3 School of Health Administration, Faculty of Health, Dalhousie University, Halifax, Canada; 4 Modeling in Health Research Center, Institute for Future Studies in Health, Kerman University of Medical Sciences, Kerman, Iran; 5 Liver and pancreatobiliary Diseases Research Center, Digestive Diseases Research Institute, Tehran University of Medical Sciences, Tehran, Iran; 6 Molecular Medicine Research Center, Hormozgan University of Medical Sciences, Bandar Abbas, Iran; 7 Health Sciences Research Center, Addiction Institute, Mazandaran University of Medical Sciences, Sari, Iran; 8 Occupational Health Research Centre, School of Public Health, Shahid Sadoughi University of Medical Sciences, Yazd, Iran; 9 Gastrointestinal and Liver Diseases Research Center, Guilan University of Medical Sciences, Rasht, Iran; 10 Health Promotion Research Center, Zahedan University of Medical Sciences, Zahedan, Iran; 11 Dept. of Neurology, Medical School, Rafsanjan University of Medical Sciences, Rafsanjan, Iran; 12 Gastroenterohepatology Research Center, Shiraz University of Medical Sciences, Shiraz, Iran; 13 Liver and Gastrointestinal Diseases Research Center, Tabriz University of Medical sciences, Tabriz, Iran; 14 Digestive Disease Research Center, Ardabil University of Medical Sciences, Ardabil, Iran; 15 Traditional and Complementary Medicine Research Center, Sabzevar University of Medical Sciences, Sabzevar, Iran; 16 Modeling in Health Research Center, Shahrekord University of Medical Sciences, Shahrekord, Iran; 17 Social Determinants of Health Research Center, Urmia University of Medical Sciences, Urmia, Iran; 18 Noncommunicable Diseases Research Center, Fasa University of Medical Sciences, Fasa, Iran; 19 Colorectal Research Center, Shiraz University of Medical Sciences, Shiraz, Iran; 20 Department of Nutrition, Faculty of Medicine, Mashhad University of Medical Sciences, Mashhad, Iran; 21 Nutrition and Metabolic Diseases Research Center, Ahvaz Jundishapur University of Medical Sciences, Ahvaz, Iran

**Correction: BMC Public Health 20, 214 (2020)**

**https://doi.org/10.1186/s12889-020-8322-8**

In the original﻿ [1] publication there was an incorrect ethic approval code. The incorrect and correct code are available in this correction article, the original article has been updated.

**Incorrect Ethics approval and consent to participate**

While each cohort center received the ethical approval from local universities, for the purpose of this study and pooling all PERSIAN data, the ethics committee of Kermanshah University of Medical Sciences approved the study (IR.KUMS.REC.1397.187).

**Correct Ethics approval and consent to participate**

While each cohort center received the ethical approval from local universities, for the purpose of this study and pooling all PERSIAN data, the ethics committee of Kermanshah University of Medical Sciences approved the study (**IR.KUMS.REC.1397.866**).
